# Anxiety-like Behavior in Female Sprague Dawley Rats Associated with Cecal Clostridiales

**DOI:** 10.3390/microorganisms11071773

**Published:** 2023-07-07

**Authors:** Tracey Bear, Nicole Roy, Julie Dalziel, Chrissie Butts, Jane Coad, Wayne Young, Shanthi G. Parkar, Duncan Hedderley, Hannah Dinnan, Sheridan Martell, Susanne Middlemiss-Kraak, Pramod Gopal

**Affiliations:** 1 The New Zealand Institute for Plant and Food Research Limited, Palmerston North 4410, New Zealand; chrissie.butts@plantandfood.co.nz (C.B.); shanthi.parkar@gmail.com (S.G.P.); duncan.hedderley@plantandfood.co.nz (D.H.); hannah.dinnan@plantandfood.co.nz (H.D.); sheridan.martell@plantandfood.co.nz (S.M.); pramod.gopal@plantandfood.co.nz (P.G.); 2 Riddet Institute, Massey University, Palmerston North 4442, New Zealand; nicole.roy@otago.ac.nz; 3 School of Food and Advanced Technology, Massey University, Palmerston North 4442, New Zealand; j.coad@massey.ac.nz; 4 Department of Human Nutrition, Otago University, Dunedin 9016, New Zealand; 5 High-Value Nutrition National Science Challenge, Auckland 1145, New Zealand; 6 AgResearch Ltd., Grasslands Research Centre, Palmerston North 4442, New Zealand; wayne.young@fonterra.com

**Keywords:** stress, anxiety, microbiome, cytokines, microbiome–gut–brain axis, Clostridiales

## Abstract

The relationship between the microbiota profile and exposure to stress is not well understood. Therefore, we used a rat model of unpredictable chronic mild stress (UCMS) to investigate this relationship. Depressive-like behaviors were measured in Female Sprague Dawley rats using the sucrose preference test and the Porsolt swim test. Anxiety-like behaviors were measured with the light–dark box test. Fecal corticosterone, cecal microbiota (composition and organic acids), plasma gut permeability (lipopolysaccharide-binding protein, LBP) and plasma inflammation (12 cytokines) markers were measured. Atypical behaviors were observed in female rats following UCMS, but no depressive-like behaviors were observed. Circulating concentrations of cytokines granulocyte-macrophage colony-stimulating factor and cytokine-induced neutrophil chemoattractant 1 were higher in UCMS-exposed female rats; plasma LBP and cecal organic acid levels remained unchanged. Our results reflect a resilient and adaptive phenotype for female SD rats. The relative abundance of taxa from the Clostridiales order and *Desulfovibrionaceae* family did, however, correlate both positively and negatively with anxiety-like behaviors and plasma cytokine concentrations, regardless of UCMS exposure, supporting the brain-to-gut influence of mild anxiety with a microbiota profile that may involve inflammatory pathways.

## 1. Introduction

Depression and anxiety episodes are very commonly preceded by stress [1]. Therefore, identifying the factors associated with the development of symptoms of anxiety and/or depression following stress allows the identification of possible avenues of prevention or treatment for depression and anxiety. The gut microbiome has been shown to be associated with emotional behaviors in rodents and humans, and it is also affected by stress [2]. The composition of fecal microbiota has also been shown to be associated with stress sensitivity or stress resilience in male Sprague Dawley rats [3]. Increased gut permeability and systemic inflammation have been shown to be associated with the increased severity of depression symptoms [4,5], and both gut permeability and inflammation can be increased by stress [6,7,8,9,10,11] and may be mediated by gut microbiota [12].

Therefore, it is plausible that individual differences in stress-induced changes in the gut microbiota composition and associated physiological effects could contribute to individual differences in stress resilience [2].

We hypothesized that stress-induced changes in behavior would be associated with changes in the gut microbiota and either alterations in short-chain fatty acids (SCFAs) with an associated increase in systemic inflammation and/or an increase in gut permeability, with an associated increase in systemic inflammation. We used the unpredictable chronic mild stress (UCMS) model to study female Sprague Dawley rats for six weeks to assess the effect of stress on behavior, gut microbiome, cecal SCFAs, gut permeability marker plasma lipopolysaccharide-binding protein (LBP), and plasma cytokines interferon-gamma (IFN-γ), cytokine-induced neutrophil chemoattractant type-1 (CINC-1), monocyte chemoattractant protein-1 (MCP-1), tumor necrosis factor-alpha (TNF-α), granulocyte-macrophage colony-stimulating factor (GM-CSF), interleukin (IL)-18, IL-12p70, IL-1β, IL-17A, IL-33, IL-1α, and IL-6.

## 2. Materials and Methods

### 2.1. Design

**Figure 1:**
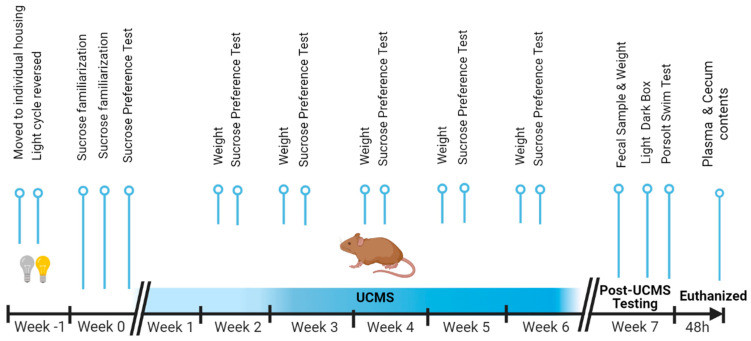
Timeline of the study.

The study included an experimental group exposed to six weeks of UCMS (UCMS group, *n* = 20) and a group not exposed to stress (control group, *n* = 10). The control group was kept in a separate room to ensure they were not exposed to any of the UCMS procedures. Behavioral tests were conducted throughout and at the end of the UCMS procedure (see Figure 1).

### 2.2. Animals

Conventional female Sprague Dawley rats (*n* = 30) were obtained in house from the animal breeding facility at the Plant and Food Research Institute, Palmerston North, New Zealand. The rats were weaned into family groups of six rats at three weeks of age. At seven weeks old, two weeks before the beginning of the study, they were separated into individual housing, and the light cycle was reversed to a 12:12 h dark: light cycle (0700/1900 h) so that behavioral tests could be conducted in their active (dark) phase. Both groups were individually housed in shoebox cages and provided with ad libitum food and water. Individual housing is stressful for rats, but it was needed to avoid sharing gut microbiota via coprophagy. The rats weighed 259 g +/− 19.4 g at the beginning of the study. Procedures were approved by the Grasslands Animal Ethics Committee (approval code 14297) in accordance with the Animal Welfare Act 1999.

### 2.3. Procedures

#### 2.3.1. Unpredictable Chronic Mild Stress Procedure

Rats were divided into the UCMS and control groups, with baseline sucrose preference counterbalanced between the two groups. The stress group was subjected to a UCMS protocol, modified from Varga et al. [13], as described below. The stress protocol comprised 10 to 12 h periods of stressors, which were rotated in varying order over six weeks. To prevent the sharing of fecal microbiota via coprophagy, cage crowding (4–6 rats per cage) was removed from the protocol. It was replaced with individual housing in cages with wire bases, under which used bedding from unfamiliar rats was placed to cause olfactory stress. The other stressors were wet bedding (250 mL water poured into bedding), stroboscopic lighting, intermittent lighting (lights on and off every two hours), and loud continuous noise (untuned radio at 90 dB), and the cage being tilted (45°). All stressors selected are commonly used in published UCMS protocols [14]. The control group was exposed to normal laboratory practice, with cage enrichment toys rotated every few days. Weekly sucrose preference testing (SPT) was conducted, and weight and coat state measurements were taken for both groups.

#### 2.3.2. Sucrose Preference Test

The method used was a two-bottle SPT over 24 h using the method of Kelly et al. [15]. In the two-bottle test, one bottle of water and one bottle of sucrose solution were provided. The bottles were stored inverted in the room for at least 6 h before each test to ensure they were not dripping, and the solution was at room temperature. The bottles were weighed at the beginning of the test, at 12 h, and after 24 h. The side of the cage the bottles were on was switched after 12 h to reduce the effects from side preference. Sucrose preference was defined using the equation: sucrose preference = (sucrose solution consumed/total fluid consumed) × 100%. The rats were habituated to the taste of sucrose solution by being given two bottles of 1% sucrose solution for 6 h two days before the baseline SPT was undertaken to prevent neophobia (fear of new food) during the SPTs.

#### 2.3.3. Coat State Measurement

Coat state was measured using the method of Ibarguen-Vargas et al. [16], with seven different body areas scored 0 for a groomed coat or 1 for an unkempt coat, with the sum of these scores being the coat state measurement. Measurements were taken once before the beginning of the UCMS protocol and at weekly weighing for six weeks in total.

#### 2.3.4. Light–Dark Box Test

Rats were tested in opaque plastic boxes (50 cm × 50 cm × 50 cm) with black, plastic-lidded inserts (25 cm × 50 cm × 50 cm). The compartments were connected via a small opening (10 cm × 10 cm). Testing began when the rats were placed in the dark compartment, and the lid was closed. Tests ran for 5 min and were filmed. Behavioral measures were manually recorded by a blinded researcher using the footage. The behavioral measures recorded were time spent in the light compartment, latency to enter the light compartment, number of transitions between compartments, number of head pokes from the dark compartment into the light compartment, and time spent in head poking, number of stretch-attend postures from the dark compartment into the light compartment, and time spent stretching, and number of rears and time spent rearing in the light compartment.

#### 2.3.5. Porsolt Swim Test

To measure stress-induced coping behaviors, the Porsolt swim test (PST) was performed. The PST method [17] was used with a modification. On both the habituation day and the following test day, the rats were placed into tanks (50 cm tall and 22 cm in diameter) filled with 30 cm of water at 25 °C for five min. After the PST, each day, the rats were removed, dried, and placed back in their home cage. The rats were filmed from the side of the cage. Behaviors were categorized using the footage by a treatment-blinded researcher. Swimming, climbing and immobile behaviors were categorized as outlined in Slattery et al. [17]. Immobility behavior is defined as the rat floating in the water and making minimal movements. Swimming behavior is when the rat is actively moving horizontally around the cylinder with forward momentum. Climbing behavior is an upward directed movement, often against the side of the cylinder. Diving is when the rat intentionally dives to the bottom of the cylinder and comes back up. Latency to immobility is defined as the first time an immobility behavior is maintained for ≥2 s, as per the protocol of Kelly et al. [15].

#### 2.3.6. Sample Collection

Fecal samples were collected in week six by placing the rats into clean cages and collecting the first two fecal pellets. They were collected in the morning. If they did not defecate within 5 min, their tails were gently lifted to induce defecation. Pellets were collected immediately, placed in sterile tubes, and frozen at −80 °C until analysis. Rats were euthanized using CO_2_ gas 48 h after the last behavioral test (PST).

Blood was withdrawn via a cardiac puncture into a lithium heparin vacuum tube. The tubes were centrifuged, and plasma was separated into aliquots and stored at −80 °C until analysis.

Cecal digesta was collected into sterile tubes, snap frozen in dry ice, and then stored at −80 °C until analysis.

#### 2.3.7. Organic Acid Analysis

Cecum organic acids (formate, acetate, propionate, isobutyrate, butyrate, isovalerate, valerate, hexanoate, heptanoate, lactate, and succinate) were analyzed using gas chromatography according to a modified method of Richardson et al. [18]. Briefly, cecum samples were defrosted and diluted 5 × with phosphate-buffered saline (10 mmol PBS; Sigma, Auckland, New Zealand) containing 2-ethyl butyric acid (Merck, Auckland, New Zealand) as an internal standard and homogenized. They were then centrifuged at 10,000× *g* for 10 min, and an aliquot of 500 µL of the supernatant was removed, and 250 µL HCL and 1000 µL diethyl ether was added. The diethyl ether phase was stored at −80 °C until analysis using a gas chromatographer (GC). Samples were again homogenized and centrifuged (IEC Micro CL 17R; Thermo Electron Corporation, Auckland, New Zealand) at 10,000× *g* for 5 min. Next, 100 µL of the upper ether supernatant layer was removed, and 20 µL of derivatizing agent (N-tert-butyldimethylsilyl-N-methyltrifluoroacetamide (MTBSTFA)) was added, and the sample was stored at −80 °C for 20 min, and then room temperature for 48 h. In a capped GC vial, 100 µL of the diethyl ether phase was derivatized with 20 µL MTBSTFA with 1% tert-butyldimethylchlorosilane (MTBSTFA + TBDMSCI, 99:1; Sigma-Aldrich, St. Louis, MO, USA) by heating it to 80 °C in a water bath for 20 min. The samples were left for 48 h at room temperature before analysis to allow complete derivatization to occur. Standards containing 2-ethylbutyric acid (5 mM) as an internal standard were prepared for derivatization alongside the samples.

Analysis was performed using a Shimadzu capillary GC system (GC-2010 Plus, Tokyo, Japan) equipped with a flame ionization detector (FID) and fitted with a Restek column (SH-Rtx-1, 30 m × 0.25 mm ID × 0.25 µm) (Shimadzu, Portland, OR, USA). The carrier gas was helium with a total flow rate of 21.2 mL/min and a pressure of 131.2 kPa. The makeup gas was nitrogen. The temperature program began at 70 °C, increased to 115 °C at 6 °C/min, with a final increase to 300 °C at 60 °C/min, and it was held for 3 min. The flow control mode was set to linear velocity; 37.5 cm/s. The injector temperature was 260 °C, and the detector temperature was 310 °C. Samples were injected (1 µL) with a split injection (split ratio: 10). The GC instrument was controlled, and data were processed using Shimadzu GC WorkStation LabSolutions Version 5.3. Organic acid quantification was assessed using the peak areas for formate, acetate, propionate, isobutyrate, butyrate, isovalerate, valerate, hexanoate, heptanoate, lactate, and succinate, relative to ethyl butyric acid. The data acquired provided a final sample result of µmol organic acid/g wet cecal digesta.

#### 2.3.8. Characterization of the Fecal Microbiota Composition

The overall microbial community composition of the cecum digesta was analyzed via 16S rRNA gene sequencing. Following the preparation of the cecum digesta for organic acids, 25 g of the pellet was used for 16S rRNA extraction using the Qiagen DNeasy Powersoil Kit isolation kit (MoBio Laboratories, Carlsbad, CA, USA) in a final volume of 50 μL. Modifications to the manufacturer’s instructions included subjecting the samples to 3 × 90 s bead beating cycles on the FastPrep-24™ 5G (MP Biomedicals, Seven Hills, Australia) at 5.5 m/s, with 5 min ice rests between cycles. DNA quality and quantity were assessed using a Nanodrop spectrophotometer (ND-1000, ThermoFisher, Waltham, MA, USA).

The samples were sent for 16S RNA sequencing at Massey Genome Service (Massey University, Palmerston North, New Zealand), where PCR was run to amplify variable regions V3-V4 of the 16S rRNA gene using barcoded fusion primers 16SF_V3 (5′-AATGATACGGCGACCACCGAGATCTACAC-barcode-TATGGTAATTGGCCTACGGGAGGCAGCAG-3′) and 16SR_V4 (5′-CAAGCAGAAGACGGCATACGAGAT-barcode-AGTCAGTCAGCCGGACTACHVGGGTWTCTAAT-3′) [19], which also contain adaptors for downstream Illumina MiSeq sequencing. Each sample was amplified with a pair of unique (8 bases) barcoded primers.

The PCR conditions used were a hold at 95 °C for 2 min, followed by 30 cycles of 95 °C for 20 s, 55 °C for 15 s, 72 °C for 5 min, finishing with a hold at 72 °C for 10 min. The PCR reagents were Invitrogen AccuPrime™ Pfx SuperMix (Cat—12344-040) (17 µL), 10 μM 16SR_V4 Primer (1 μL), 10 μM 16SF_V3 Primer (1 μL) and 1 µL normalized sample (5 ng/µL). The PCR library clean-up kit used was an Invitrogen SequalPrep Normalization Plate Kit (Thermo Fisher Scientific, Waltham, MA, USA). Eighteen µL of the PCR product was used for the library clean up, and the elution volume was 12 µL. A Qubit DNA High-Sensitivity assay was used to measure the library concentration, and a Bioanalyzer DNA High-Sensitivity assay was used for library sizing. The amplicons were pooled in equal molarity, and 16S rRNA gene sequencing was performed via an Illumina MiSeq 2 × 250 base paired-end run.

#### 2.3.9. Fecal Corticosteroid Metabolites

Fecal corticosteroid metabolites are representative of serum corticosteroid concentrations from around 14–16 h earlier and are commonly used as a non-invasive measure for adrenocortical activity in animals [20,21]. It also avoids the confounding increase in corticosterone level from the stress of acute sampling methods, and therefore, measures the baseline stress levels [20]. Corticosterone was extracted using the following method. First, fecal samples were defrosted and freeze dried at 4 °C for 12 h, and then crushed into powder using a metal spatula. Next, absolute ethanol was added at a concentration of 1 mL ethanol per 0.1 g fecal material (dry weight) and shaken for 30 min at 800 mot/min. Then, they were centrifuged at 10,000× *g* for 15 min, and the supernatant was removed and frozen at −80 °C. Fecal corticosterone concentrations in the supernatant were analyzed in duplicate using an Eliza kit (ENZO AD1-901-071, Enzo Life Sciences, Farmingdale, NY, USA) according to the manufacturer’s instructions using a 1:500 dilution of the extracted corticosterone solution. The coefficient of variation (CV%) was accepted at less than 10. Calculated values are reported as nanograms of corticosterone per milliliter.

#### 2.3.10. Plasma Cytokines and Lipopolysaccharide-Binding Protein Concentrations

Plasma was thawed at room temperature (18 °C). Plasma cytokines, interferon-gamma (IFN-γ), cytokine-induced neutrophil chemoattractant type-1 (CINC-1), monocyte chemoattractant protein-1 (MCP-1), tumor necrosis factor-alpha (TNF-α), granulocyte-macrophage colony-stimulating factor (GM-CSF), interleukin (IL)-18, IL-12p70, IL-1β, IL-17A, IL-33, IL-1α, and IL-6, were studied. The cytokines were measured in duplicate using a bead-based Legendplex assay (Kit 740401, Biolegend, San Diego, CA, USA) in accordance with the manufacturer’s instructions. A dilution of 1:4 was used. Analyses were performed using a BD FACSverse flow cytometer (BD Biosciences, San Jose, CA, USA). Data were analyzed using Legendplex V8.0 software (BioLegend) and are presented as pg/mL.

The plasma concentration of LBP was measured in duplicate using an ELISA kit (ENZO, Cat. #ALX-850-305) in accordance with the manufacturer’s instructions. A dilution of 1:20 was used. Absorbance was measured at 450 nm using FLUOStar Optima^®^ (BMG Labtech, Mornington, VIC, Australia). The coefficient of variation (CV %) was accepted at less than 10. Calculated values are reported as nanograms of LBP per milliliter.

### 2.4. Statistical Analysis

The coat state, sucrose preference, and sucrose intake were compared between the UCMS and control groups using two-way factorial repeated measures ANOVA. Sucrose preference scores were log transformed before analysis to stabilize the variance. Normality was determined with a visual inspection of residual plots. Differences between individual time points were compared using least significant differences (LSD) at a level of 5%. Results of the PST, plasma cytokine concentrations, body weight, fecal corticosterone and cecal organic acid concentrations were compared between the UCMS and control groups using a two-tailed independent *t*-test with a probability value of 5%, following a normality check using a Shapiro–Wilk test. Correlations were undertaken using Spearman’s Rank Coefficient. Due to the large number of comparisons, a correction for a false discovery rate of 5% was performed using the Benjamini–Hochberg procedure. In addition, scatterplots were visually checked for outliers and a visual correlational relationship for both groups. Due to the high collinearity of data, partial least squares (PLS) regression was used to reduce the dimensionality and assess if any dependence relationship was apparent at the population level. Microbial taxa at the species level, SCFA, cytokines, and LBP were included as the independent variables, and behavioral variables were the dependent variables. Variables with 50% or more values at a lower or upper threshold value were removed. The thresholds were zero count for microbiota (before conversion to relative abundance); no time spent in behavior during behavioral tests; or below the detection limits for cytokines, LBP, and organic acids. In addition, variables with a skewness greater than 1.8 or kurtosis greater than 3.8 were also removed. These analyses were conducted using the statistical software package GENSTAT, version 19.1.0.21390 (VSN International (2022), Genstat for Windows 22nd Edition, VSN International, Hemel Hempstead, UK). The microbial sequence data analysis was performed using QIIME 2, version 2019.10 [22]. The sequences were quality checked, denoised, and chimeric sequences were removed in a DADA2 step [23] (trimmed by removing 10 bases from the sequences and truncating at base number 235). This output had a total of 572,949 reads and 1685 features (taxa) for 30 samples, with a minimum frequency (read) of 12,463. These data were used for the analysis of alpha diversity (Observed_OTUs, Chao1, Shannon Index and Simpson Index) using no rarefaction, and beta diversity (Bray–Curtis, weighted, and unweighted Unifrac distances methods with sampling depth set at 12,000) [24] conducted using phyloseq within R [25], (version 3.5.0 23 April 2018). For differential abundance analysis, the operational taxonomic units (OTUs) that have a frequency of less than 0.1% of the mean sample depth were removed to account for possible Illumina sequencing errors, and taxa not seen more than two times in 10% samples to remove OTU with small mean and large CV values were filtered out. The taxonomy associated with the SILVA reference database was used [26] (version 132, 99%, released on 13 December 2017). Taxa were analyzed for differential abundance using the Analysis of Compositions of Microbiomes with Bias Correction (ANCOM-BC) [27] method using the statistical program, R.

## 3. Results

### 3.1. Sucrose Preference Test

**Figure 2:**
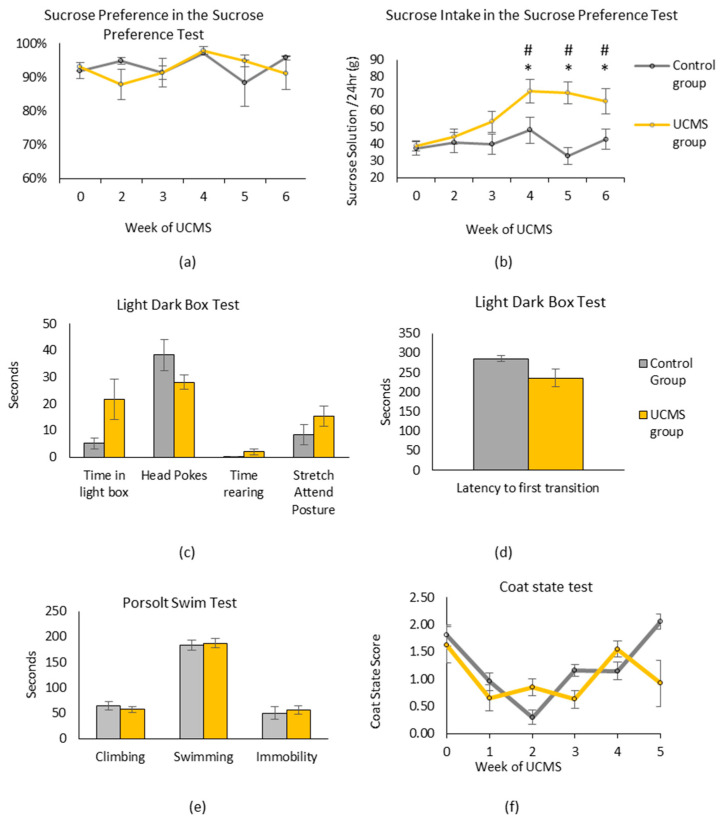
Results of behavioral testing unpredictable chronic mild stress in the UCM group and the control group. (**a**) Sucrose preference, measured in weekly 24 h sucrose preference tests (SPT) over six weeks of UCMS was not altered by time or exposure to UCMS, but (**b**) the total sucrose solution intake during the same tests increased in the UCMS group over six weeks. (**c**,**d**) Following six weeks of studying UCMS rats and control rats, no significant differences in individual scores in the light–dark box test were found. (**e**) There was no difference in scores in the Porstolt swim test between groups following six weeks of UCMS. (**f**) There were also no differences between the two groups in the coat state test. Data are presented as mean + SEM; control group, *n* = 10; UCMS group, *n* = 20. * indicates *p* < 0.05 between UCMS group and control group. # indicates *p* < 0.05 between timepoint and baseline.

The sucrose preference (Figure 2a) did not significantly change over time (F(5, 139 = 1.16, *p* = 0.331), and there was no difference in sucrose preference between the UCMS and control groups (F(1, 28) = 0.05, *p* = 0.826). The sucrose intake (Figure 2b), however, was higher overall in the UCMS group, (F(1, 28) = 5.75, *p* = 0.023). Post hoc analysis using LSD showed this was driven by a significant difference between the groups from weeks 4 to 6. Due to an increase over time in the UCMS group, but not in the control group, there was a significant effect of time (F(5, 139) = 9.81, *p* ≤ 0.001) and a significant interaction between time and UCMS (F(5, 138) = 4.13, *p* = 0.004). Adjusting for weight did not significantly affect the results of the analysis of covariance (F(1, 27) = 5.54, *p*= 0.026).

### 3.2. Light–Dark Box Test

None of the light–dark box test measures showed significant differences between the UCMS and control groups (Figure 2c,d).

### 3.3. Porsolt Swim Test

The results show no difference between the control and UCMS groups in regard to either immobility (t(28) = 0.39, *p* = 0.700), swimming (t(28) = 0.26, *p* = 0.798), climbing (t(28) = 0.78, *p* = 0.442), or fecal output (t(28) = 0.98, *p* = 0.334) (Figure 2e).

### 3.4. Coat State Measurements

There was no difference in the coat state scores (Figure 2f) between the UCMS group and the control group (F(1, 28) = 1.63, *p* = 0.212). However, there was an effect of time on the coat state scores (F(5, 5) = 11.96, *p* ≤ 0.001), with the coat state scores decreasing in both groups in weeks 1–3 after the instigation of the UCMS. There was also a treatment x time interaction (F(5, 5) = 5.73, *p* ≤ 0.001), with the pattern of change differing, particularly in week five.

### 3.5. Weight

There was no difference in weight (t(28) = −0.08, *p* = 0.935) between the UCMS group (mean 311.1 g +/− 5.4 g SEM) and control group (mean 310.2 g +/− 9.9 g SEM) following six weeks of UCMS.

### 3.6. Fecal Corticosterone

The fecal corticosterone concentrations measured in week six showed no difference (U(10, 20) = 73.0, *p* = 0.248) between the UCMS group (mean 28.6 ug/mL +/− 2.6 μg/mL SEM) and the control group (mean 47.0 μg/mL +/− 11.7 μg/mL SEM).

### 3.7. Plasma Lipopolysaccharide-Binding Protein (LBP)

There was no difference in plasma LBP (t(28) = −1.6, *p* = 0.12) between the UCMS group (mean 0.273 +/− 0.013 SEM) and control group (0.244 ± 0.019 SEM).

### 3.8. Cytokines

**Figure 3:**
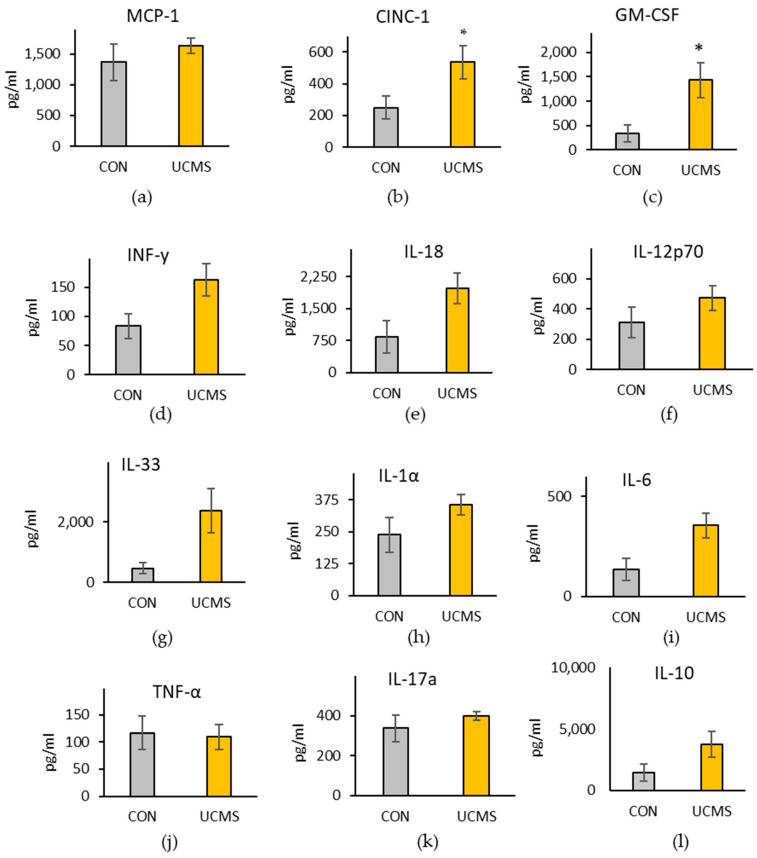
Plasma cytokine concentrations. In female Sprague Dawley rats, following six weeks of unpredictable chronic mild stress (UCMS), a pattern of increased pro- and anti-inflammatory markers was observed compared with those of the control group (CON). Graphs shows the measured cytokines which were (**a**) monocyte chemoattractant protein-1 (MCP-1), (**b**) cytokine-induced neutrophil chemoattractant type-1 (CINC-1), (**c**) granulocyte-macrophage colony-stimulating factor (GM-CSF), (**d**) interferon-gamma (IFN-γ), (**e**) interleukin (IL)-18, (**f**) IL-12p70, (**g**) IL-33, (**h**) IL-1α, (**i**) IL-6, (**j**) tumor necrosis factor-alpha (TNF-α), (**k**) IL-17A, (**l**) IL-10. Data are presented mean +/− SEM; control group, *n* = 10; UCMS group, *n* = 20. * indicates a significantly different value from the control group at *p* < 0.05.

There was an overall pattern of increased plasma cytokine concentrations in the UCMS group compared with those of the control group (Figure 3a–l). The significantly increased cytokines were CINC-1 (t(28) = 2.25, *p* = 0.033), and GMC-CSF (t(28) = 2.67, *p* = 0.013). IL-1β was below the detection limit in 29 out of 30 samples.

### 3.9. Organic Acids

No difference was found in the concentration of individual organic acids between the control and UCMS groups (see Table 1).

### 3.10. Cecal Microbiota Composition

**Figure 4:**
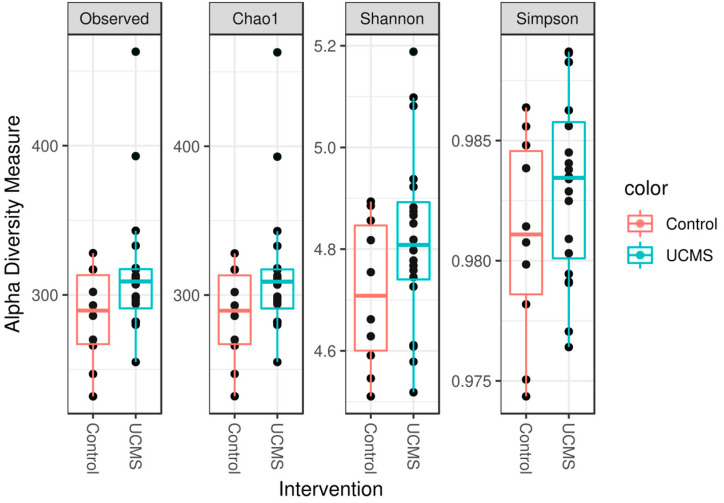
The alpha diversities (measured via four indexes: observed, Chao1, Shannon, and Simpson) of the cecal microbiota of Sprague Dawley rats did not differ after six-week exposure to unpredictable chronic mild stress (UCMS) between the UCMS group or the control group.

**Figure 5:**
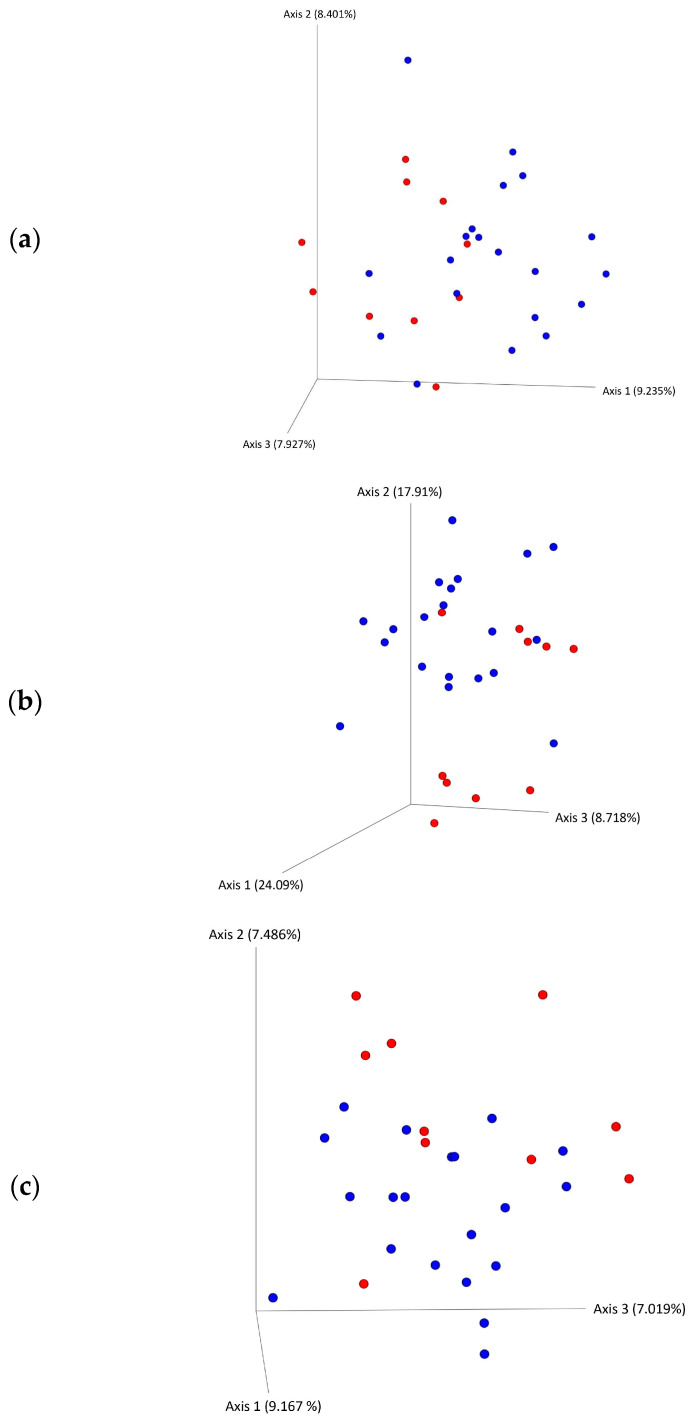
Beta diversity PCA plots of the cecal microbiota of Sprague Dawley rats after six-week exposure to unpredictable chronic mild stress (shown in blue) and control rats (shown in red). PCA plots show (**a**) Bray–Curtis distances, (**b**) weighted unifrac distances, and unweighted unifrac distances (**c**).

The alpha diversity measures (observed, Chao1, Shannon, and Simpson) were not significantly different between the UCMS group and CON group (Figure 4). The beta diversity differed between the UCMS and control groups (Figure 5) when assessed using the Bray–Curtis distance, weighted, and unweighted Unifrac distances.

**Figure 6:**
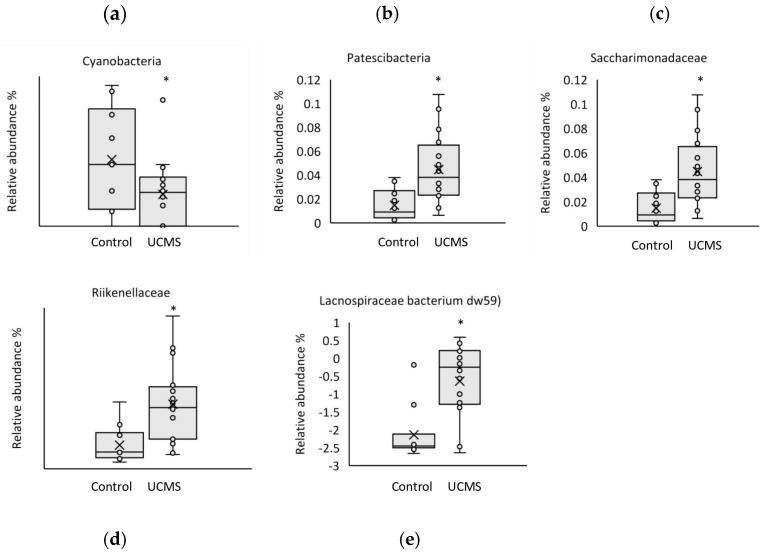
Relative abundance of cecal microbiota taxa identified via 16S rRNA next generation sequencing. Female Sprague Dawley rats exposed to six weeks of unpredictable chronic mild stress (UCMS) showed differences between groups in several taxa (**a**–**e**) than the control group did. Box and whisker plots show relative abundance of the microbial taxa for each rat, with the minimum value, first quartile, median, third quartile, and maximum value of each group shown. *p* = phylum; f = family; sp = species. * indicates significant difference at *p* value < 0.05. Data are presented mean +/− SEM; UCMS group, *n* = 20; control group, *n* = 10.

Several taxa differed between the UCMS and control groups (Figure 6). The phylum, Cyanobacteria (w = 2.536, *p* = 0.011), showed a lower relative abundance, and the phylum, Patescibacteria (w = 3.318, *p* = 0.001), showed a higher relative abundance in the UCMS group compared with that in the control group. The relative abundance of the families, *Saccharimonadaceae* (w = 2.986, *p* = 0.003) and *Rikenellaceae* (w = 3.029, *p* = 0.002), and that of the species, *Lachnospiraceae bacterium* DW59 (w = 4.570, *p* < 0.001), were also higher in the UCMS group. There were no changes for any genera. Species within *Lactobacillus* and *Bifidobacterium*, genera with known an association with emotional behavior, showed no significant difference between groups.

### 3.11. Partial Least Squares Regression between Behavior and Biological Variables

No relationship between the behaviors and any biological variables was found via PLS regression analysis. The significance testing of dimensions using Osten’s F-test showed that the predicted residual sum of squares (PRESS) for the PLS models was higher than the original data were, indicating over-fitting. This result indicates that there was no population-level association between these variables.

### 3.12. Correlation Analysis between Cecal Microbiota and Behavior

Spearman’s rank correlations were completed between the behavioral variables, cecal organic acids, plasma cytokines, fecal corticosterone, plasma LBP, and with the relative abundance of cecal microbiota at the species level in the UCMS and control groups separately, and with the two groups combined. Correlations were identified for each analysis using a *p* value of 0.05. The significant *p* value was then adjusted to *p* = 0 to correct for a false discovery rate (FDR) of 5%. Following FDR correction, several significant correlations remained between the behavioral variables, cytokines, and microbial species (see Table 2).

### 3.13. Associations between Behavior, Cecal Microbial Taxa, SCFAs, Lipopolysaccharide-Binding Protein and Cytokines

Associations were found between behavior and cecal microbiota. In the UCMS group, a negative correlation was identified between an unspecified bacterium of the *Lachnospiraceae NK4A136* group and the time spent in a stretch-attend posture. A positive correlation was observed in the UCMS group between the number of head pokes in the light–dark box test and the species, *Oscillibacter* sp. 1–3. No significant correlations were identified in the control group. When both groups were combined and used in the analysis, negative correlations were found between an unclassified species from Clostridiales Family XIII with two behaviors in the light–dark box test (time spent in the light box and the number of transitions) and an unspecified species in the *Ruminococcus* genus with the number of fecal pellets excreted in the PST. All the bacteria that were found to correlate with behavior were in the order Clostridiales of the Firmicutes phylum.

Positive correlations were observed between three microbes and cytokines (see Table 2). Strong positive correlations were found in the control group between an uncultured bacterium in the genus, *Ruminiclostridium,* with plasma cytokines, IL-6 and CINC-1. Moderate positive correlations were found in the UCMS group between uncultured bacterium in the genus, *Ruminococcaceae NK4A214*, and plasma IL-1α and MCP-1. Strong positive correlations were found in the control group between an uncultured bacterium in the *Desulfovibrionaceae* family of the Proteobacteria phylum and plasma cytokines, GM-CSF, IL-1α, IL-6, IL-10, and IL-18. Interestingly, two of these microbes were within the same taxa, which correlated with the behavioral variables.

The initial individual correlation analysis showed numerous weak correlations between cecal organic acids and plasma cytokines, but no individual significant correlations were identified following FDR correction. No correlation was found between any cecal organic acids and plasma LBP even before correction with the FDR.

**Table 2:** Table of r values of fecal microbiota species that significantly correlated with behaviors or plasma cytokines. Correlation analysis was undertaken on female Sprague Dawley rats, in those exposed to unpredictable chronic mild stress (UCMS), those kept as controls, and a third analysis with all rats combined. Correlations were considered significant when *p* < 0.01 following correction for a false discovery rate of 5%.

Taxonomy						Behavioral Variables					Cytokines						
						LDB Light Time (s)	LDB Transitions (n)	PST Fecal Pellets (n)	LDB SAP (s)	LDB Head Pokes (n)	GM-CSF	IL-10	IL-18	IL-1α	IL-6	CINC-1	MCP-1
Firmicutes																	
	Clostridia																
		Clostridiales															
			*Family XIII*														
				Unspecified		−0.63 (combined)	−0.65 (combined)										
					Unspecified												
			*Lachnospiraceae*														
				*Lachnospiraceae NK4A136 group*					−0.77 (UCMS)								
					Uncultured bacterium												
			*Ruminococcaceae*														
				*Ruminiclostridium*											0.94 (Con)	0.92 (Con)	
					Uncultured bacterium												
				*Oscillibacter*						0.74 (UCMS)							
					*Oscillibacter* sp. 1–3												
				*Ruminococcaceae NK4A214 group*										0.75 (UCMS)			0.76 (UCMS)
					Uncultured bacterium												
				*Ruminococcus*				−0.647 (combined)									
					Unspecified												
Proteobacteria																	
	Deltaproteobacteria																
		Desulfovibrionales															
			*Desulfovibrionaceae*														
				Uncultured							0.93 (Con)	0.93 (Con)	0.95 (Con)	0.95 (Con)	0.92 (Con)		
					Uncultured bacterium												

Note: GM-CSF, granulocyte-macrophage colony-stimulating factor; IL, interleukin; CINC-1, cytokine-induced neutrophil chemoattractant 1; MCP-1, monocyte chemoattractant protein-1; TNF, tumor necrosis factor; UCMS, unpredictable chronic mild stress exposed group; Con, control group; combined, combined UCMS and control group combined.

## 4. Discussion

Adult female Sprague Dawley rats were exposed to six weeks of UCMS to test if stress-induced changes in the cecal microbiota composition, cecal organic acids, gut permeability, and inflammation were associated with stress-induced behavioral changes. The exposure to UCMS resulted in atypical behavioral changes. In contrast to the typical results obtained using this model, the UCMS exposed rats did not show a decrease in sucrose preference (a marker of depression-like behavior) during or after exposure to UCMS. There was also no difference between the UCMS and control groups in terms of behavioral measures in the PST, LDB, or coat state measurements. Instead, they showed an increase in sucrose solution intake over time and a stronger pattern of lower anxiety-like behavior in the light–dark box test compared to that of the control group (but no differences in individual measures).

The meaning of an increase in sucrose intake in the SPT is unclear. Several other rat studies have found similar changes in behavior following stressor exposure [28,29,30,31,32] and also over time without exposure to stress [28]. It could be a different expression of anhedonia. A reduction in anhedonia does not automatically cause a decrease in a certain behavior, simply that the hedonic pleasure associated with that behavior is reduced. Inter-individual differences dictate whether the individual performs that behavior less often due to the lack of enjoyment or performs that behavior more often to obtain the same level of enjoyment previously experienced. Anhedonia has, for example, been associated with binge eating in people [33]. It is also plausible that the increased sucrose intake in the UCMS rats could be due to increased time awake during the light phase, when they would usually spend most of their time sleeping, because of a disrupted circadian rhythm. A disruption in the circadian rhythm and associated activity times and levels can be caused by stress [34], but also the UCMS procedure included altering the light schedule (lights on during the dark phase or alternating light and dark every two hours during the dark phase), which is also likely to have caused circadian disruption.

### 4.1. Plasma Inflammation Markers and Gut Permeability

An increase in plasma cytokines CINC-1 and GM-CSF concentrations and a pattern of increase in several other cytokine concentrations were seen in the UCMS group compared with the control group. Corticosterone can be immunosuppressive, particularly for those with chronic stress [35]; so, this result is consistent with the corticosterone concentrations being higher in the control group. There was no difference in the plasma concentration of gut permeability marker, LBP, between groups, nor correlations between plasma cytokines, plasma LBP, or behaviors in the UCMS group.

The current study hypothesized that a stress-induced increase in gut permeability (and therefore, systemic lipopolysaccharide (LPS) exposure) would cause systemic inflammation, which would be associated with stress-induced behavior. Inflammation as a potential mediator of depression is not well understood, but one mechanism is thought to cause the alteration of tryptophan metabolism [36]. Key enzymes can divert the metabolism of tryptophan towards the kynurenine pathway, which produces more neurotoxic/excitatory neuro-molecules. Some of these enzymes (e.g., indole-2,3-dioxygenase (IDO)) are stimulated by LPS, IFNs, TNF- α, IL-1β, and IL-6 [37]. In the current study, the concentrations of LBP (a proxy for LPS levels) and TNF-α were not increased, and that of IL-1β was below the detection limit. The IFN-y and IL-6 levels showed only a small non-significant increase.

Therefore, it is possible that the lack of typical depression-like behavior in the UCMS rats was because the inflammation profile as assessed by the LBP, and cytokine levels did not cause alteration to the kynurenine pathway.

### 4.2. Differences in Cecal Microbiota between Groups

The cecal microbiota compositions differed between the control and UCMS groups. The beta diversity (measure of dissimilarity between the samples) was altered following UCMS, but the alpha diversity (measure of richness and evenness of microbiome within a sample) was not. The UCMS group showed lower relative abundance of the phylum, Cyanobacteria, and higher relative abundance of the phylum, Patescibacteria, families, *Saccharimonadaceae* and *Rikenellaceae*, and the species, *Lachnospiraceae bacterium DW59* compared with those of the control group. The cecal organic acid levels did not differ from those of the control group, suggesting that changes to the microbiota structure did not result in major changes to the metabolic activity of the cecal microbiome.

There are some similarities in the changes in microbial taxa in the current study with those in previous stress studies. An increase in *Saccharimonadaceae* in colonic bacteria has been previously observed in mice exposed to a combination of chronic restraint stress and UCMS [38]. Increases in the abundance of *Rikenellaceae* have been found in mice following acute restraint stress [39] and chronic dark stress [40]. Rikenellaceae also seem to be associated with behavior. A reduction in *Rikenellaceae* levels following antibiotic treatment in BALB/c and NIH Swiss mice occurred alongside a decrease in anxiety-like behavior [39,41]. *Rikenellaceae* has also been associated with emotionality in humans. In children, an undefined genus of *Rikenellaceae* was positively correlated with a high rating of activity and/or high-intensity pleasure among boys and positively associated with fear among girls [42]. Also, the genus *Alistipes* belonging to the *Rikenellaceae* family has been associated with depression [43,44].

The finding of a decrease in the level of the phylum, Cyanobacteria, is novel. This finding has not been found to differ in terms of abundance in other stress studies, including those on maternal separation stress in rats [45,46,47] or in mice exposed to acute social stress [48], acute restraint stress [39], chronic restraint stress [49,50,51,52], chronic grid floor stress [53], chronic mild stress [54,55,56], chronic mild stress [57], or five weeks of behavioral testing [58]. Emerging research has shown a higher abundance of Cyanobacteria in people with gastrointestinal, metabolic, and respiratory issues and Graves’ disease [59]. This shows only an association rather than cause; however, the administration of mass-cultured Cyanobacteria was shown to have a hypoglycemic effect when administered to diabetic rats [60]. This suggests that stress-induced changes in the cecal microbiota of this phylum could have a physiological effect.

### 4.3. Correlations between Different Bacteria and Behaviors

Several correlations in the present study were identified between individual cecal microbes and behaviors. The individual correlations between several cecal microbes and behaviors indicate that there was an association with emotional behavior. However, the PLS analysis results, which compares data in a group rather than individual measures, did not show the same correlation. This indicates that the role of individual bacteria may be more important than the overall composition.

Negative correlations were found between exploratory/behavioral activation measures (time spent in light box, transitions between light and dark, and time spent in the stretch-attend posture) and unspecified species in Clostridiales Family XIII and genus, *Lachnospiraceae* NK4A136 (of the family *Lachnospiraceae*). A positive correlation was found between exploratory behavior (head pokes) and the species, *Oscillibacter* sp. 1–3 (of the family *Ruminococcaceae)*. An unspecified bacterium in the *Ruminococcus* genus (of the family *Ruminococcaceae)* was also negatively associated with the number of fecal pellets expressed in the PST.

These same three microbial families (*Lachnospiraceae*, *Ruminococcaceae*, and *Clostridiales* Family XIII) have been linked to anxiety-like behaviors in previous research. A higher relative abundance of these families was observed in tail-biting pigs compared with that of the non-tail biters [61], and the abundance of taxa from the family *Ruminococcaceae* was also higher in hens that had higher rates of feather pecking [62]. Both tail biting in pigs and feather pecking in chickens are stress-induced behaviors [61,62]. The abundance of *Clostridiales* Family XIII has been positively associated with anxiety scores in people suffering from mood disorders [63]. *Clostridiales* Family XIII has also been associated with depressive-like behaviors. A species in the *Clostridiales* Family XIII, UCG 001, has been shown to be decreased in rats susceptible to developing pain-induced anhedonia [64].

### 4.4. Mechanisms

The mechanisms for how these microbes could affect anxiety have not yet been established. The correlations between anxiety-like behavior and the cecal microbial abundance in this study do not prove causality, but it is plausible that a causal relationship could exist. In rats with depression and the reduced abundance of a species in *Clostridiales* Family XIII UCG 001, depression-like behaviors were able to be transferred to antibiotic-induced pseudo germ-free mice with a fecal microbial transplant [64]. This indicates that the microbiome was caused depressive-like behaviors. In rats bred for an anxious phenotype, an antibiotic (minocycline) treatment caused an increase in the abundance of *Clostridiales* Family XIII, alongside decreased depressive-like behavior (immobility in the FST) and microglial numbers, although there were no differences in anxiety-like behaviors [65].

The gut microbiota plays an important role in the production of serotonin in the gut. Gut microbial-derived organic acids, SCFAs, are known to stimulate hosts’ gut enteroendocrine cells to produce serotonin [66,67], and *Clostridium* species may be key microbes that have this effect [66,68]. *Ruminococcaceae* and *Lachnospiraceae* have been associated with the carbohydrate metabolism [69] and produce butyrate [70]. Whether the serotonin levels in the gut affect serotonin levels in the brain is not yet clear.

The negative correlation between the relative abundance of *Ruminococcus* spp. and the number of fecal pellets excreted by rats during the PST, which is representative of stress-induced changes in lower gut motility, suggests an association with the sympathetic nervous system. It is possible that the *Ruminococcuss* spp. can influence the sympathetic nervous system, but it is also possible that the relationship between Clostridiales and anxiety could simply be due to alterations in the gut environment (increased motility and differences in pH) due to increased sympathetic activity in the enteric nervous system. No correlation was found between any cecal bacteria relative abundances and fecal corticosterone concentrations; however, the fecal and cecal samples were collected at different timepoints six days apart.

### 4.5. Correlations between Circulatory Inflammation Markers and Cecal Microbiota

It seems the correlational relationship between the cecal microbiota and behavior could also be associated with inflammation. In both groups, species in the *Ruminococcaceae* family also correlated with plasma cytokines, MCP-1 and IL-1α. MCP- has previously been reported to correlate with *Ruminococcaceae* in mice [71].

Whether the cecal microbes affected inflammation or vice versa (or were changed by a third variable) is uncertain. Some research suggests that changes in systemic immune function can alter the gut microbiota [72,73]. Here, there was no correlation between any cytokines and behavior, which suggests that whatever the relationship is between the microbiota and inflammation, it is not a mechanism that causes differences in behavior.

The positive correlation between an uncultured bacterium in the *Desulfovibrionaceae* family in the control group only and plasma GM-CSF, IL-10, IL-18, IL-1α, and IL-6 concentrations is consistent with the fact that taxa from the family, *Desulfovibrionaceae*, are endotoxin-producing bacteria, which are inflammatory.

### 4.6. Whether the Correlations Are Stress-Dependent Is Uncertain

Neither the microbes nor behaviors that were found to correlate matched those which differed between the UCMS and control groups. This finding suggests that the correlational relationships are not stress-dependent. Another possibility, however, is that both groups were stressed, and the stress levels were too similar between groups to show accurate stress-induced changes between groups. Several factors suggest that this could be the case. No difference in body weight or fecal corticosterone was found between the group undergoing UCMS. Typically, stressed animals have a reduced body weight and increased fecal corticosterone following stress e.g., [74]; however, there is some variability in the response to stress, and it can depend on the intensity and duration of the stress [75,76], or other factors such as social status [77].

The UCMS protocol in this study used stressors at the milder end of what many protocols are reported to use [14]. In previous studies on Sprague Dawley rats, in which depressive-like behaviors developed in the rats, more intensive stressors, such as restraint stress, cold stress, overcrowding, swim stress, and food and water deprivation, were used [78,79,80]. It is possible that the stressors used may have been too mild, allowing the UCMS rats to become habituated to the UCMS stressor and experience a low level of stress or no chronic stress. It is also plausible that the control group experienced chronic stress at a similar level as the rats in the UCMS group did. The rats were kept in different rooms, and there may have been unaccounted factors in the control group room that caused stress. There are several variables in animal care that could contribute to stress, including differences in sounds, smells, temperatures, handling, and lighting [81,82]. Isolation stress due to single housing is also plausible. It is well known that single housing causes isolation stress and can increase anxiety-like and depressive-like behaviors [83]. However, the UCMS group was also singly housed. It is possible that the isolation stress of single housing was ameliorated in the UCMS group due to novel stressors of UCMS causing either psychological adaptation (reduced stress during acute behavioral test stressors) or physiological adaptation (periodic acute increases in corticosterone, allowing negative feedback to occur). The reason for using singly housed rats for both the control and UCMS groups was to prevent the exchange of fecal microbiota via coprophagy. The co-housing of genetically modified mice with conventional mice has been shown to cause the transmission of fecal metabolites that can influence the physiology of conventional mice [84]. There are numerous studies that have successfully used singly housed rats as a control group for stress intervention research [13,85,86,87,88].

Because of the uncertainty of the stress levels of both the UCMS and control groups, comparisons between the groups should be interpreted with caution, particularly directionality. However, they are reported and discussed here because as more research is undertaken in the microbiome–gut–brain axis field, the implications of these results may become clearer.

### 4.7. Lack of Typical Depressive-like Behavior Could Be Due to Lack of Gut Permeability

The implication of stress-induced changes in microbes in the current study being similar to those of previous research, without concomitant behavioral changes, is that microbial and behavioral changes due to stress may be independent and not causal. It is also plausible that these microbes can induce depressive-like behavior in rodents, but we did not focus on this in this study because there was no increase in gut permeability due to LPS. This finding has been previously reported. A study on adult male C57Bl/6J mice reported increased sucrose intake and minor changes in their fecal microbiota at the family and genus levels following three weeks of psychosocial stress, but there was no change in depressive-like behavior. While the stressor increased gut permeability to fluorescein isothiocyanate–dextran, low plasma LBP levels showed that bacterial translocation did not occur due to the maintenance of the mucus layer [30].

### 4.8. Limitations

For logistical reasons, only one sex could be used in the current study. Female rats were chosen because depression occurs with higher prevalence in women compared with that in men [89]. There is high neurological, hormonal, and genetic variability between males and females, and research on male rodents may not translate well to female humans [90,91]. Because of the reported differences in responses to stress due to sex, the results from this study cannot be generalized to males. Additionally, the estrous cycles of the rats were not measured during the study. It is possible that differences in the estrous cycle between the groups could explain the differences between the control and UCMS groups. However, the correlation results are unlikely to be affected by differences in the estrous cycle.

A change in diet can affect the microbiome; so, there is some chance that the difference in sucrose intake during the sucrose preference test could have caused a difference in microbiota between the groups. However, the difference was measured only over one 24 h period per week and constituted less than 0.04% of their (typical) weekly calorie intake. Additionally, there was no association observed between sucrose intake and microbiota. Differences in overall food intake could also cause a difference in the gut microbiota; while food intake was not measured, there was no difference in weight or weight gain between the groups, and therefore, it was likely that they had a similar intake. The effect of diet cannot be ruled out; however, the change in the cecal microbiota was more likely to be a mixture of long-term effects from the chronic UCMS stressor and a response to acute stress.

## 5. Conclusions

Following six weeks of UCMS, female Sprague Dawley rats showed an increase in sucrose intake over time, rather than a decrease in sucrose preference, suggesting that the rats showed an atypical response to stress. They also showed a larger change in cecal microbial beta diversity and plasma inflammatory cytokines, CINC-1 and GM-CSF, compared with that of the control group.

The plasma inflammatory markers had an overall pattern of increased concentration, which was not associated with increased gut permeability or anxiety-like or depressive-like behaviors. However, several correlations were observed between anxiety-like behaviors and microbial relative abundance in the order, Clostridiales, along with correlations between microbes and cytokines. These results support the growing body of evidence that the gut microbiota is associated with anxiety. This novel result also suggests that bacteria in the order, Clostridiales, could be important in this relationship. Further research is needed to determine the direction of causality.

## Figures and Tables

**Table 1:** Organic acid concentrations in cecal digesta. There was no difference between concentrations in female Sprague Dawley rats following six weeks of unpredictable chronic mild stress (UCMS) and those of the unexposed control group. Concentrations are in µmol Organic Acid/g wet weight. Mean and standard errors are reported.

	Control			UCMS		
Organic Acid	Mean		SEM	Mean		SEM
Formate	<0.30	+/−	0.00	<0.30	+/−	0.00
Acetate	80.17	+/−	6.28	79.26	+/−	2.75
Propionate	10.92	+/−	0.54	9.68	+/−	0.34
Isobutyrate	0.52	+/−	0.07	0.44	+/−	0.04
Butyrate	31.64	+/−	3.34	34.44	+/−	1.38
Isovalerate	0.26	+/−	0.05	0.22	+/−	0.02
Valerate	1.08	+/−	0.03	1.02	+/−	0.03
Hexanoate	1.41	+/−	0.11	1.51	+/−	0.08
Heptanoate	<0.10	+/−	0.00	<0.10	+/−	0.00
Lactate	0.33	+/−	0.08	0.31	+/−	0.03
Succinate	0.38	+/−	0.11	0.30	+/−	0.02

## Data Availability

The data that support the findings of this study are available from the corresponding author, upon reasonable request.
